# Redox Regulation of the Actin Cytoskeleton in Cell Migration and Adhesion: On the Way to a Spatiotemporal View

**DOI:** 10.3389/fcell.2020.618261

**Published:** 2021-01-28

**Authors:** Emre Balta, Johanna Kramer, Yvonne Samstag

**Affiliations:** Section Molecular Immunology, Institute of Immunology, Heidelberg University, Heidelberg, Germany

**Keywords:** L-plastin, cofilin, oxidation, actin, T cell, spatiotemporal, migration, adhesion

## Abstract

The actin cytoskeleton of eukaryotic cells is a dynamic, fibrous network that is regulated by the concerted action of actin-binding proteins (ABPs). In particular, rapid polarization of cells in response to internal and external stimuli is fundamental to cell migration and invasion. Various isoforms of ABPs in different tissues equip cells with variable degrees of migratory and adhesive capacities. In addition, regulation of ABPs by posttranslational modifications (PTM) is pivotal to the rapid responsiveness of cells. In this context, phosphorylation of ABPs and its functional consequences have been studied extensively. However, the study of reduction/oxidation (redox) modifications of oxidation-sensitive cysteine and methionine residues of actin, ABPs, adhesion molecules, and signaling proteins regulating actin cytoskeletal dynamics has only recently emerged as a field. The relevance of such protein oxidations to cellular physiology and pathophysiology has remained largely elusive. Importantly, studying protein oxidation spatiotemporally can provide novel insights into localized redox regulation of cellular functions. In this review, we focus on the redox regulation of the actin cytoskeleton, its challenges, and recently developed tools to study its physiological and pathophysiological consequences.

## Introduction

### Actin Cytoskeleton and ABPs

The actin cytoskeleton is important for maintaining the shape and structure of eukaryotic cells, as well as for such essential processes as cell migration, cell polarity, intracellular or extracellular trafficking, cell-cell interactions, and cell division. These processes are regulated by ABPs through the supply of globular actin (G-actin) for polymerization, nucleation of new filaments, depolymerization and severing, capping, branching, and formation of actin bundles [reviewed in Samstag et al. (2003)].

Actin is a 42-kDa globular protein that can be reversibly polymerized into filaments (F-actin). The length of the filaments is controlled by capping proteins and by actin-depolymerizing and -severing proteins like ADF-1 and cofilin (Samstag et al., 2013). The organization of higher-order structures, such as filopodia, invadopodia, lamellipodia, stress fibers, and microvilli requires actin bundles. Actin-bundling proteins such as plastins form F-actin into parallel or antiparallel arrays. These bundles provide the actin structures with structural stability and elasticity (Morley, 2012; Stevenson et al., 2012). Overall, spatiotemporal regulation of ABPs enables rapid rearrangement of the actin cytoskeleton in response to stimuli, and leads to formation of the right structures in the right place and at the right time (Winder and Ayscough, 2005; Davidson and Wood, 2016). Studies in recent years have shown that PTMs on ABPs dictate the responses of the cytoskeleton. In this review, we highlight the importance of redox regulation of ABPs and exemplify emerging tools to study this regulation in the future.

### ROS Sources and Protein Thiol Oxidation

Reactive oxygen species (ROS) are produced in mitochondria, the endoplasmic reticulum (ER), and peroxisomes, or by specialized enzymes such as nicotinamide adenine dinucleotide phosphate (NADPH) oxidases (NOXes). There are seven NOX isoforms: NOX1–5 and DUOX1–2 (Hampton et al., 1998). These multi-subunit enzymes catalyze the generation of ${\text{O}}_{2}^{-}$ from NADPH and O_2_ and are primarily localized at the plasma membrane and at the membrane of organelles (Brandes et al., 2014).

Cell types differ in their capacity to produce and detoxify ROS. Elevated ROS levels, termed a pro-oxidative micromilieu, have been implicated in various pathophysiological conditions, including aging and cancer (Jones, 2006; Harris and DeNicola, 2020). Cells use various antioxidant systems to maintain the balance of ROS. These include thioredoxins, important oxidoreductases that are e.g., highly upregulated in several tumor types to compensate for pro-oxidative settings (Raffel et al., 2003; Samaranayake et al., 2017). When the intracellular redox balance is disturbed and shifts toward a pro-oxidative micromilieu, toxic levels of oxidation on protein thiols, DNA, and lipids can result in cellular senescence and death (Sies and Cadenas, 1985; Jones, 2006). In small quantities, ROS, particularly H_2_O_2_, are important signal carriers acting through reversible cysteine oxidation on several proteins (Yang et al., 2007).

Cysteine thiol oxidation can change a protein's functions, stability, interaction partners, and localization, as well as affect the presence and degree of other PTMs. Thus, redox-sensitive cysteines serve as switches that ultimately interconnect biological functions, allowing the control of cellular signaling and functions (Jones, 2010; Go and Jones, 2013). In this context, several protein tyrosine phosphatases (Cho et al., 2004; Yang et al., 2007; Behring et al., 2020), cell cycle regulatory proteins (Wu and Momand, 1998; Burch and Heintz, 2005), growth factors, and actin cytoskeleton-regulating proteins (Tang et al., 1999; Lassing et al., 2007; Klemke et al., 2008; Hung et al., 2011; Parri and Chiarugi, 2013; Fremont et al., 2017) are known to be regulated by thiol switches (see below).

### Redox Regulation of Cell Migration and Adhesion

Cells migrate in two- and three-dimensional environments by mesenchymal and amoeboid migration modalities, and a mixture of both, depending on the physical barriers, the topology and composition of the extracellular matrix (ECM), the type and degree of chemotaxis, and other cellular constituents of the environment (Yamada and Sixt, 2019). A highly dynamic and elastic actin cytoskeleton and rapid formation of cellular extrusions are fundamental to all types of cell migration.

The direction of moving cells is guided by growth factors and chemokines. Their binding to corresponding receptors initiates an array of signaling events leading to the recruitment and activation of ABPs (Blanchoin et al., 2000; DeMali et al., 2002; Yilmaz and Christofori, 2010). Mesenchymal cell migration comprises several coordinated steps that primarily depend on actin dynamics: actin polymerization and depolymerization; cell adhesion; and actomyosin contraction cycles. Actin polymerization at the leading edge of cells initiates formation of invadopodial and filopodial structures in which the interaction of integrins with the ECM results in further recruitment of ABPs such as actin-bundling proteins (Blanchoin et al., 2000; Huttenlocher and Horwitz, 2011). This contributes to the maturation of actin-based cellular protrusions. Formation of focal adhesions at the leading edge and resolving at the rear is critical for the establishment of polarity and forward movement of the cells (Yamada and Sixt, 2019). Focal adhesions represent molecular assemblies that anchor cells to the ECM via integrins and are hubs for signaling events. Degradation of the ECM by matrix metalloproteinases in invadopodial or podosomal structures paves the way at the cell front and contractile structures made up of actomyosin fibers deliver forces in order to push forward the rest of the cell body and rear (uropod) (Yamada and Sixt, 2019). The forward movement of the cell requires detaching at the cell rear which is primarily mediated by actin-severing proteins like cofilin. Cofilin also mediates the actin flow which is crucial for amoeboid cell migration. Particularly, lymphocytes in tissues make use of this mode of migration. It is characterized by a rounded cell morphology with cellular protrusions called blebs (Gaylo et al., 2016; Yamada and Sixt, 2019).

During cell migration, ROS can be generated by intracellular sources or exogenously in the surrounding micromilieu (Weinberg et al., 2019). The type, concentration, and location of ROS can differently influence cell migration and adhesion through the oxidation of signaling proteins, through oxidation of actin itself, or through the oxidation of ABPs, such as cofilin and L-plastin (LPL).

**Figure 1:**
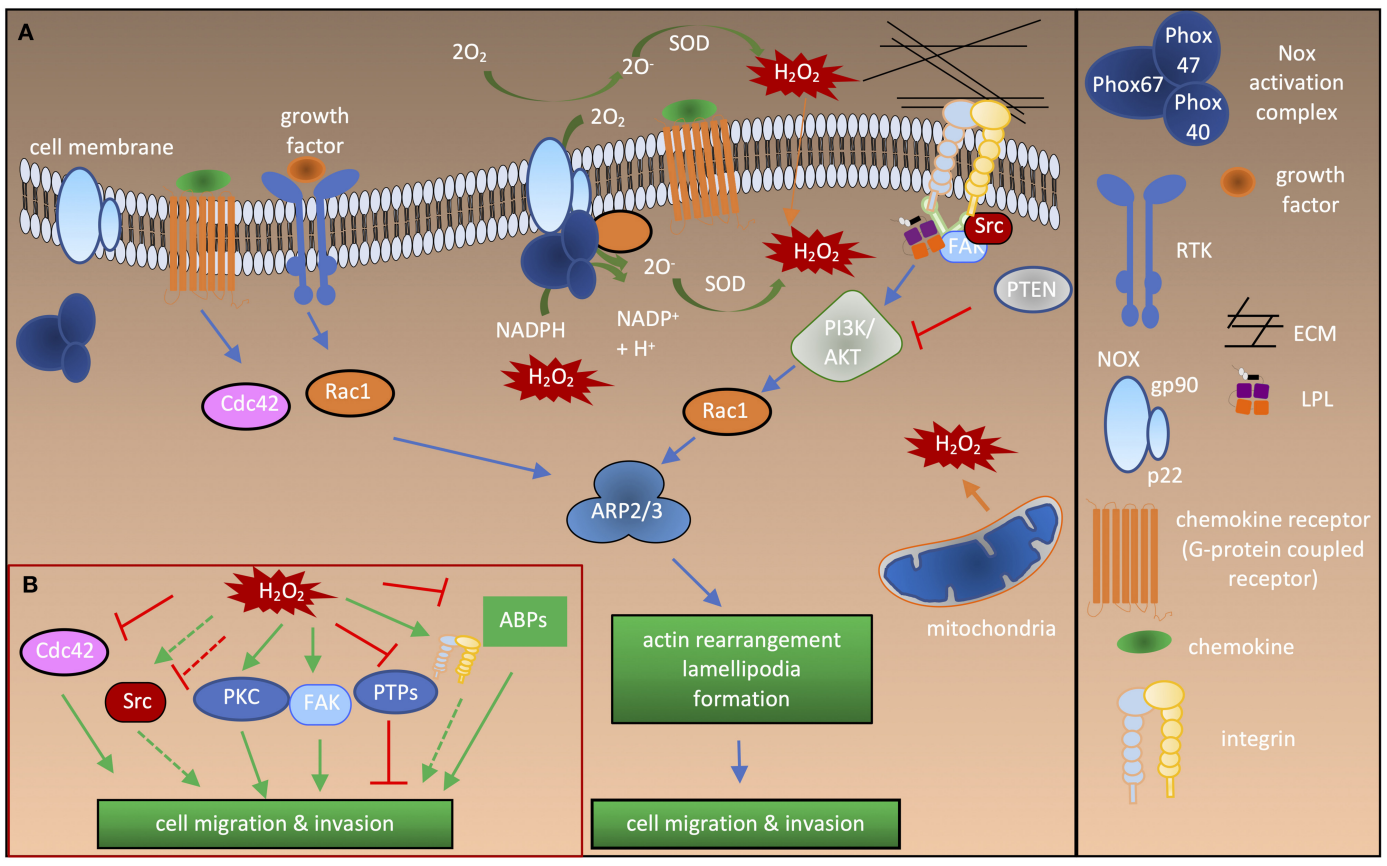
Redox regulation of proteins involved in cell activation and migration. **(A)** Cdc42 and Rac1 activation after stimulation by growth factors or chemokines leads to recruitment of the ARP2/3 complex, thereby inducing actin branching and polymerization at the leading edge. Bidirectional interactions of integrins with the ECM and intracellular interactions with adaptor molecules, such as talin and LPL lead to recruitment of Src and FAK kinases. This results in directional actin polymerization and formation of cellular extrusions. ROS are generated by NOXes either intracellularly or extracellularly in response to growth factor or chemokine stimulation. Different NOXes have different activation complexes and Rac1 activity is necessary for activation of NOX1-3. Extracellular ROS radicals (${\text{O}}_{2}^{-}$) are converted to H_2_O_2_, which enters the cell through the plasma membrane or via aquaporins (not shown). ROS are also produced by mitochondria. Note that NOX2 is depicted as an example in the figure. **(B)** Influence of ROS on the signaling molecules and ABPs involved in cell migration and adhesion. Solid green arrow, activation of protein; dashed green arrow, “potential” activation of protein; solid red lines, inhibition of protein activity; dashed red lines, “potential” inhibition of protein activity.

Accumulating evidence suggests that, physiologically, low levels of ROS are produced by NOXes in response to growth factor and chemokine stimulation in various cell types. For example, fibroblast growth factor was shown to induce NOX1 activity which promoted the migration of fibroblasts (Schröder et al., 2007). Similarly, hepatocyte stimulation by epidermal growth factor (EGF) induced NOX activity which was shown to be important for cell spreading and migration (Flinder et al., 2011). Pathophysiologically, in solid tumors ROS produced by NOXes were reported to be critical for epithelial-to-mesenchymal transition, tumor cell migration, and invasion (Tobar et al., 2010; Kim and Cho, 2014). In particular, overexpression of NOX4 induced through TGF-β has been implicated in migration of epithelial (Tobar et al., 2010), breast cancer (Boudreau et al., 2012), and melanoma cell lines (Ribeiro-Pereira et al., 2014). Similarly, other NOXes are reported to be critical for progression of various cancer types (Konate et al., 2020). Blockade of endogenous ROS production in migrating cells has provided strong evidence that NOX-induced ROS are central to cell migration (Heo et al., 2008; Tobar et al., 2010; Tamborindeguy et al., 2018). However, how NOXes are induced by these stimulations remains largely elusive, as does how ROS produced by NOXes are involved in thiol switches on specific proteins. Downstream of growth factor or chemokine stimulation during migration or adhesion, integrins cluster at focal adhesions, signaling molecules, such as protein kinases, and protein tyrosine phosphatases (PTPs) are recruited, and actin polymerization and rearrangement take place; these processes are also regulated by ROS (Figure 1A).

### Redox Regulation of the Actin Cytoskeleton

**Figure 2:**
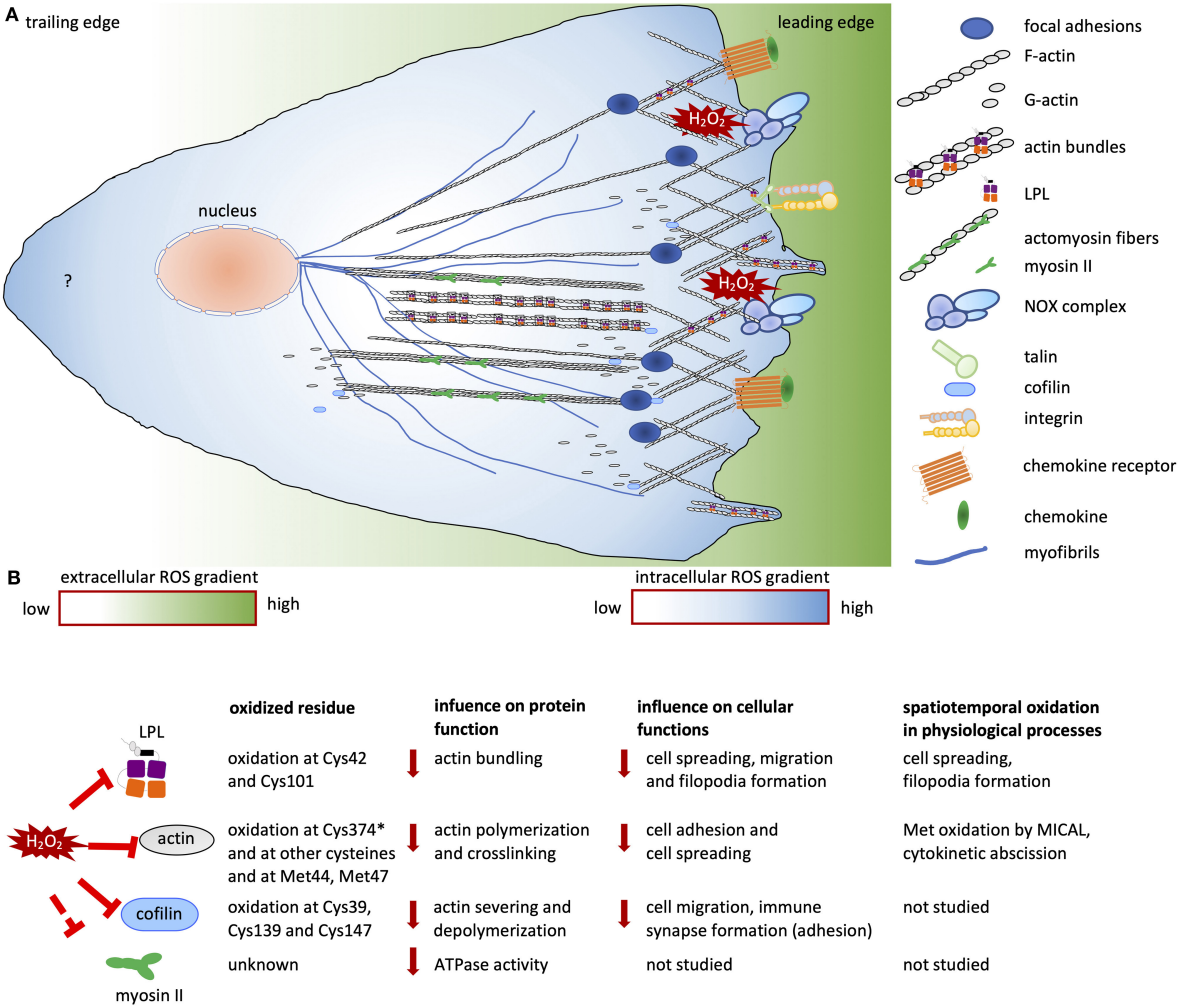
Redox regulation of cell migration. **(A)** Migrating cells establish polarity in response to chemokine and growth factor stimuli. At the leading edge, branched networks called lamellipodial protrusions and focal adhesions are formed. Filopodial extensions are formed by actin bundles. The cells contain an intracellular ROS gradient owing to localized ROS production resulting from NOX activity at the leading edges of migrating cells. At the rear, the sources and role of ROS are not known (indicated by a question mark). The intracellular ROS gradient is depicted in blue and white; dark blue and white indicate high and low ROS concentrations, respectively. The ROS gradient in the surrounding micromilieu is depicted in green and white; these colors indicate high and low extracellular ROS concentrations, respectively. **(B)** List of redox-regulated ABPs and actin, showing oxidized residues, the influence of oxidation on protein function and the consequent cellular functions, and data on spatiotemporal oxidation. “*” indicates the most sensitive cysteine on actin among other oxidized cysteine residues. Solid red lines indicate inhibition of protein activity; dashed red lines indicate “potential” inhibition of protein activity. Solid red arrows indicate downregulation of protein and cellular functions.

During cell adhesion and migration, ECM-integrin complexes are formed, bringing the cytoskeleton and other signaling proteins to the sites of new cytoskeletal assembly (Figure 1A). ROS regulate the actin cytoskeleton at several stages. Transcription factors including NF-κB, AP1, NRF2, HIF1-α (Staal et al., 1995; Kim et al., 2010) and signaling enzymes [PI3K/Akt and mitogen-activated protein kinase (MAPK)] can be indirectly regulated by ROS (Koundouros and Poulogiannis, 2018; He et al., 2019). Thus, ROS can influence the expression of various genes, including those encoding ABPs (Clarkson et al., 2002). The second regulation level is the direct oxidation of kinases and phosphatases, leading to their activation or deactivation, and thereby controlling the phosphorylation state and activity of ABPs (Figure 1B). In recent years, it has become clear that direct oxidation of actin and ABPs also has an important role in regulating actin cytoskeletal rearrangements (Figures 2A,B).

### Redox Regulation of Signaling Molecules Orchestrates Actin Cytoskeletal Dynamics

#### Integrins

Integrins are transmembrane proteins that link the cell cytoskeleton to the ECM and bidirectionally transmit signals between cells and their environment, termed inside-out or outside-in signaling (Hynes, 2002). Integrins are heterodimers composed of α-subunits (18 types) and β-subunits (8 types). They bind to components of the ECM as well as to other receptors on neighboring cells. Intracellularly, integrins are connected to the actin cytoskeleton by adaptor proteins including talin and tensin (Calderwood and Ginsberg, 2003; Kechagia et al., 2019). Rezende et al. showed that two cysteines of integrin α7β1 formed a disulfide bridge after H_2_O_2_ treatment in vascular smooth muscle cells, and that this oxidation increased integrin binding to laminin (de Rezende et al., 2012). A follow-up study revealed thiol switches on two cysteines in the hinge region of the α7 chain, resulting in a high-ligand-binding conformation and thus regulating integrin binding to laminin, cell morphology, and migration (Bergerhausen et al., 2020).

#### Kinases

Focal adhesion kinase (FAK), a non-receptor tyrosine kinase orchestrates signaling cascades in the focal adhesions of migrating cells. It carries an integrin-binding domain and two proline-rich sequences that bind to SH2 domain-containing proteins (Mitra et al., 2005). FAK is activated by autophosphorylation at Tyr397 upon integrin binding to promote cell motility and adhesion. In protrusions of migrating cells, FAK signaling to downstream GTPases regulates changes in actin and microtubule structures. In this context, FAK phosphorylates Rho-family GTPase-activating proteins and their guanine nucleotide-exchange factors, as well as ABPs (Mitra et al., 2005). ROS-induced phosphorylation at various tyrosine residues enhances the kinase activity of FAK (Ben Mahdi et al., 2000; Basuroy et al., 2010); this was recently shown to be associated with increased cell survival (Ribeiro-Pereira et al., 2014). However, it is not yet known whether the increased phosphorylation of FAK is due to inhibition of a phosphatase or activation of a kinase. Importantly, a study demonstrated that focal adhesion turnover can be regulated by ROS (Datla et al., 2014). The authors showed elevated ROS in focal adhesions of migrating vascular smooth muscle cells. An siRNA-mediated knockdown of NOX4 or of its regulator Poldip2 prevented focal adhesion stability. In light of their findings, authors implicated the importance of ROS at focal adhesions and its regulatory influence on proteins, such as RhoA GTPases and FAK (Datla et al., 2014).

Similar to FAK, various protein kinase C (PKC) family proteins are critical to the phosphorylation of ABPs. Redox-dependent activation of PKCs can occur via direct regulation of redox-sensitive cysteines, or via ROS-dependent production of lipid intermediates or ROS-induced calcium regulation [reviewed in Steinberg (2015)].

Src kinases are another family of kinases with critical roles in actin cytoskeletal rearrangements. Src kinases contain SH3 and SH2 domains and a catalytic domain that autophosphorylates Tyr residues. Src is primarily found in its inactive conformation; dephosphorylation of the autophosphorylated Tyr sites is required for its activation. Importantly, Src kinases have been reported to be oxidized at certain cysteine residues at the catalytic site; this disrupts autophosphorylation, thereby activating the Src kinases (Knock and Ward, 2011). A study of Cys245Ala and Cys487Ala mutants indicated that oxidation at these cysteines was critical for Src activity, and thus for regulation of cell invasion capacity and anchorage-dependent growth (Giannoni et al., 2005). However, in many other cellular systems direct or indirect effects of ROS on Src kinase activity could not be differentiated since both activation (Heppner et al., 2018) and inactivation (Tang et al., 2005; Kemble and Sun, 2009) of Src kinases have been reported.

#### Rho GTPases

Binding of chemokines to RTKs activates Rho family of GTPases, such as Rac1, Cdc42, and RhoA. The cycling between GDP-(inactive) and GTP-bound (active) states modulates the interaction of Rho GTPases with cellular targets during physiological processes, such as migration and adhesion.

In particular, Rho GTPases regulate recruitment and activation of the ARP2/3 complex, leading to F-actin polymerization and branching (Figure 1). Rac1, Cdc42, and Rho GTPases were reported to be regulated by ROS. Interestingly, Rac1 can regulate ROS production and is itself regulated by ROS [reviewed in Hobbs et al. (2014)]. It is evidenced that NOX1, NOX2, and NOX3 activation requires a complex comprising active Rac1 for electron transport from NADPH to O_2_ (Hordijk, 2006). Rac1 also interacts with redox-modulating enzymes such as SOD1. The latter was proposed to activate Rac1/NOX in a redox-dependent manner (Harraz et al., 2008). ROS can induce the exchange of GDP from Rac1 leading to its activation. This seems to be mediated by direct Rac1 oxidation on Cys18 at the catalytic site as a Cys-Ser mutant did not show any activation in response to H_2_O_2_ treatment (Heo and Campbell, 2005). While the specific redox modification of Rac1 was previously only associated with the formation of lamellipodia (Hobbs et al., 2014), Rac1-mediated ROS production by NOXes has been attributed to several functions including cell migration (Myant et al., 2013; Tolbert et al., 2019).

#### Protein Tyrosine Phosphatases

PTPs regulate intracellular signaling by RTKs, integrins, and cytokine receptors. They dephosphorylate several proteins of cytoskeletal signal transduction pathways (Wu et al., 2005; Li et al., 2014). PTPs contain a motif with a highly acidic catalytic cysteine residue, whose nucleophilic attack on a targeted phosphotyrosyl residue results in its dephosphorylation (Zhang and Dixon, 1994). The catalytic cysteine, which has a low pKa value, is also highly susceptible to oxidation. As a consequence, PTPs are transiently oxidized and inactivated. Well-known redox-regulated PTPs include PTEN and PTP1B (Salmeen et al., 2003; Tonks, 2005; Chen et al., 2009; Schwertassek et al., 2014).

Binding of growth factors to RTKs activates the PI3K/AKT signaling pathway which is pivotal to cell growth and survival. Activated PI3K mediates conversion of PIP_2_ to PIP_3_ which further activates downstream kinases such as AKT. As opponent of PI3K, PTEN, a plasma membrane lipid phosphatase, converts PIP_3_ into PIP_2_. Thereby, it inhibits cell growth, survival, and cell migration and acts as a tumor suppressor. In this context, lack of PTEN in glioblastoma (Davidson et al., 2010), in gastric cancer (Ma et al., 2017), and in other cancers (Coronel-Hernandez et al., 2019; Hu et al., 2019; Zhang et al., 2020) was correlated with enhanced migration and invasion. Several studies revealed inhibition of the catalytic activity of PTEN by ROS thereby allowing prolonged signaling for cell survival, proliferation, and migration. PTEN oxidation generally enhances PI3K/AKT signaling resulting in cell type- and context-dependent functional consequences (Wu et al., 2013; Kim et al., 2018). Importantly, in tumor cells PTEN oxidation promotes tumor progression (Shen et al., 2015). Another well-known oxidized PTP is PTP1B, a regulator of insulin signaling and cellular metabolism. However, PTP1B oxidation was associated with both tumor promoting and inhibiting functions (Lessard et al., 2010; Xu et al., 2019) and requires further elaboration.

### Redox Regulation of Actin and ABPs

#### Actin Oxidation

Actin has three isoforms, and all six cysteines of β/γ-actin and five cysteines of α-actin have been reported to be oxidized [reviewed in Wilson et al. (2016); Xu et al. (2017)]. Cys374 is the most critical of these redox-sensitive cysteines which can form an intramolecular disulfide bridge with Cys285 or an intermolecular disulfide bridge with Cys374 of another actin molecule (Lassing et al., 2007; Farah et al., 2011). Oxidation at Cys374 slows down the polymerization and stability of F-actin (DalleDonne et al., 1999). Another study showed that Cys374 oxidation induces actomyosin disassembly, and thus contributes to a contraction of the cytoskeleton during cell spreading and stress fiber formation (Fiaschi et al., 2006). In addition, S-glutathionylation of actin at Cys374 seems to be important for stress fiber formation, and for the spreading capacity of cells (Dalle-Donne et al., 2003; Fiaschi et al., 2006).

Further findings on thiol modifications at different cysteine residues of actin suggest different consequences depending on the cell type, and concentration and type of ROS (DalleDonne et al., 1995; Shartava et al., 1995; Moldovan et al., 2000; Wang et al., 2001; Fiaschi et al., 2006; Lassing et al., 2007; Thom et al., 2008; Farah et al., 2011). In several studies, non-physiological concentrations of exogenous ROS (mM range) were utilized which mostly diminished actin assembly. Contrarily, low concentrations of ROS were reported to positively influence actin polymerization. In this regard, an early study showed that blockade of NOXes in endothelial cell lines prevented G-actin incorporation into growing F-actin suggesting that ROS production by NOXes is critical for F-actin assembly (Moldovan et al., 2000). Similarly, blockade of NOXes downmodulated actin stress fiber formation and migration of tumor cell lines providing evidence for a positive role of ROS for localized actin polymerization and dynamics (Auer et al., 2017; Tamborindeguy et al., 2018). However, to the best of our knowledge, even though localized ROS production by NOXes during cell migration was elucidated (Kaplan et al., 2011; Tamborindeguy et al., 2018), there is no literature showing localized oxidation of actin on cysteine residues during physiological processes such as cell migration.

Actin is also regulated by oxidation at Met44 and Met47 through molecules interacting with CasL (MICAL) proteins (Hung et al., 2011; Grintsevich et al., 2016). Oxidation by MICALs diminishes inter-actin contacts, resulting in enhanced F-actin disassembly, diminished actin polymerization, and increased monomeric actin concentrations in cells (Grintsevich et al., 2017). MICAL1 specifically mediates oxidation of F-actin, which enhances the binding of cofilin to filaments. This, in turn, increases actin filament severing by cofilin and subsequent actin depolymerization (Grintsevich et al., 2016). Physiologically, localized MICAL1 functions were shown to be critical for cell division (Fremont et al., 2017). Pathophysiologically, its expression was directly linked to cell migration and invasion in breast cancer cells (Deng et al., 2018) and in melanoma cells (Loria et al., 2015).

#### Myosin II Oxidation

Myosin II motor protein is expressed in almost all cells and is divided into two categories: non-muscle and muscle myosin. Myosin II is critical for cell adhesion, migration, and division. The force that is generated by myosin II ATP hydrolysis facilitates actomyosin contractions in migrating cells. There is limited evidence regarding the redox regulation of myosin II. Initially, rat myocardial myosin II was shown to be S-glutathionylated (Passarelli et al., 2008). The myosin II homolog in protists was found to be oxidized at Met394 after H_2_O_2_ treatment, which reduced its actin-activated ATPase activity (Moen et al., 2014). A study by Fiaschi et al. showed that integrin-engagement during adhesion of fibroblasts led to ROS production. A following mass spectrometric analysis revealed that myosin II was more oxidized in adherent cells than in round cells. Further investigation showed a diminished interaction between non-muscle myosin II and actin in spreading cells suggesting a role of myosin II redox regulation for actin cytoskeletal rearrangements (Fiaschi et al., 2012). However, this phenomenon needs to be further elaborated. Moreover, none of these studies focused on specific oxidation of cysteine residues of human myosin II. A direct correlation between involvement of myosin II oxidation and its cellular functions requires identification and characterization of its redox-sensitive cysteines.

#### Gelsolin Oxidation

Gelsolin participates in actin-remodeling by sequestering actin monomers and by severing, capping, and nucleating F-actin. It is expressed abundantly in all cell types and exists as two isoforms located intracellularly and as a secreted form (Feldt et al., 2019). Human cytoplasmic gelsolin contains five cysteine residues. In the secreted protein, two of these five cysteine residues are forming disulfide bridges (Wen et al., 1996). An early study showed that gelsolin can prevent cytochrome c release from mitochondria and inhibit apoptosis (Koya et al., 2000). Moreover, elevated gelsolin expression is linked to increased intracellular superoxide levels, promoting the invasive capacity of colon cancer cells (Tochhawng et al., 2016). A recent study further revealed an increase in translocation of cytosolic gelsolin to mitochondria and a decrease in extracellular/plasma gelsolin when oxidative phosphorylation in mitochondria is dysfunctional (Garcia-Bartolome et al., 2020). Taken together, while gelsolin is regulated by ROS at the expression level, its direct redox regulation and particular functional consequences need to be elaborated.

#### Cofilin-1 Oxidation

Cell migration requires dynamic rearrangements of the actin cytoskeleton. Cofilin is a key molecule mediating actin dynamics and cell migration. Cofilin severs actin filaments, providing free barbed ends that can be used for the formation of new actin filaments or for depolymerization (Samstag et al., 2003). Cofilin-1 is expressed in non-muscle cells and is activated by dephosphorylation on Ser3 (Moriyama et al., 1996; Nagaoka et al., 1996; Nebl et al., 1996). Its activity is also controlled by thiol modifications on its cysteines (Cys39, Cys80, Cys139, and Cys147) (Klemke et al., 2008; Samstag et al., 2013). Under pro-oxidative conditions, Cys139 is modified to sulfonic acid (Cys-SO_3_H), and Cys39 and Cys80, which are buried inside the molecule, are likely to form an intramolecular disulfide bridge. Thereby, cofilin-1 loses its ability to dismantle F-actin, with consequent increases in F-actin stability and net actin polymerization. In T cells, this results in stiffening of the actin cytoskeleton, which can diminish T cell migration and cell-cell interaction, namely immune synapse formation between T cells and antigen-presenting cells (Klemke et al., 2008; Samstag et al., 2013). Excessive H_2_O_2_ exposure leads to mitochondrial translocation of cofilin-1, followed by necrotic-like programmed cell death (Wabnitz et al., 2010a). Conversely, a reducing microenvironment, such as that provided by antigen-presenting dendritic cells, prevents cofilin-1 oxidation and renders cofilin-1 insensitive to inactivation by phosphatidylinositol 4,5-bisphosphate thereby promoting T cell activation (Schulte et al., 2013).

#### LPL Oxidation

L-plastin (LPL) is an actin-bundling protein which is physiologically expressed in hematopoietic cells and ectopically expressed in malignantly transformed tumors of non-hematopoietic origin (Pacaud and Derancourt, 1993; Park et al., 1994; Klemke et al., 2007). LPL is specifically localized to sites of actin polymerization including invadopodia (Van Audenhove et al., 2016), podosomes (Zhou et al., 2016), filopodia (Delanote et al., 2010; Schenk et al., 2017), lamellipodia, stress fibers, the cell cortex, focal adhesions, and cell-cell interaction zones (Wabnitz et al., 2010b, 2016). In addition to the known enhanced activity of LPL resulting from phosphorylation on Ser5 (Shinomiya et al., 2007; Wabnitz et al., 2007, 2010b), Balta et al. showed that LPL is regulated by thiol oxidation at Cys101 and Cys42 residues, which could be reverted by thioredoxin 1 (Balta et al., 2019). In line with these data, a global analysis of cysteine thiols modified by allicin, an organosulfur compound obtained from garlic, showed that LPL was one of the top five most abundant allicin-bound proteins in Jurkat leukemia cells (Gruhlke et al., 2019). LPL oxidation diminished its actin-bundling capacity and dependent cellular functions, including cell migration and invasion. Generation of a new sensor (LPL-roGFP-Orp1) allowed spatiotemporal analysis of LPL oxidation in tumor cells. This unraveled that LPL oxidation occurred primarily at the cell periphery. It attenuated peripheral actin dynamics and particular cellular functions, such as cell spreading and filopodia formation (Balta et al., 2019).

## Discussion

As described above, ROS induce oxidation of actin, LPL, and cofilin, with additive diminishing effects on cell migration and invasion, as oxidation changes the F-actin structure, inhibits actin-bundling by LPL, and prevents dynamic actin reorganization by cofilin. Signaling molecules are also regulated by thiol modifications, thereby influencing actin cytoskeletal reorganization. Moreover, global application of ROS undoubtedly leads to oxidation of many different proteins. However, the effects of the oxidation of individual proteins on individual cellular functions remained largely unknown.

The first steps toward understanding redox regulation of cysteine thiols and the consequent changes in cellular functions involved mutation of individual cysteines in cellular proteins and studying the respective functional effects in transfected cells under control and pro-oxidative conditions. These initial studies highlighted the principal role of a given protein oxidation in cellular functions, usually under non-physiological ROS conditions.

Protein oxidation was linked to particular cellular subcompartments, such as mitochondria, peroxisomes, or the ER, where it is involved in important processes including protein and lipid biosynthesis (Ushio-Fukai, 2006; Kaplan et al., 2011; Bechtel et al., 2020). However, spatiotemporal protein oxidation can also take place throughout the cytoplasm or at certain parts of the cell membrane through localized ROS production or a localized absence of antioxidant systems, respectively. Yet, only in a handful of studies the spatiotemporal regulation of cysteine oxidation under physiological conditions was investigated (Grintsevich et al., 2017; Tsutsumi et al., 2017; Balta et al., 2019). Recently, the development of new tools has facilitated study of this phenomenon, providing insights into the redox regulation of cellular functions. In this context, a recent study found that localized oxidation of actin by MICAL1 led to localized depolymerization of actin filaments, which is critical for cytokinetic abscission (Fremont et al., 2017). NOX2 activity at the leading edge of migrating endothelial cells was also shown to be required for directional cell migration (Ushio-Fukai, 2006; Kaplan et al., 2011). Along the same lines, using a dimedone-based proximity ligation assay (PLA), specific protein oxidation was clearly detectable in the vicinity of NOXes (Tsutsumi et al., 2017). However, apart from these findings, there is limited evidence for localized ROS production or the absence of antioxidant systems during physiological processes in cells.

Studying spatiotemporal oxidation of individual ABPs is a novel and promising strategy to understand the physiological and pathophysiological redox regulation of cell migration and other cell functions. Using a dimedone-based PLA and an LPL-linked ROS sensor, Balta et al. demonstrated that spatial LPL oxidation within actin-based cellular extensions was likely to result both from low levels of antioxidants and an elevated accumulation of pro-oxidative molecules at cellular extensions. Importantly, finding spatiotemporally occurring oxidation sites of LPL also enabled a specific focus on cellular actin-based functions, e.g., actin bundling, in which LPL oxidation is critically involved (Balta et al., 2019).

These findings further suggest that spatiotemporal oxidation of ABPs or actin may have a major role in the regulation of actin-based cellular processes at the cell periphery during physiological processes such as cell migration. The methods applied could also be used to investigate spatial oxidation of many other proteins functioning in cellular extensions. Thus, fusion of the ROS sensor roGFP-Orp1 or dimedone-based PLA with other potentially oxidized ABPs or signaling molecules could be used to decipher whether they are locally oxidized, either due to their proximity to NOXes or due to the local absence of antioxidant systems.

Finally, a dysfunctional cytoskeleton resulting from oxidation of ABPs may have an important role in cancer immunology. As tumor-specific T cells require a highly dynamic actin cytoskeleton in order to infiltrate solid tumors, a pro-oxidative tumor environment and the resulting oxidations on LPL, cofilin, or actin, and potentially other ABPs in T cells might inhibit their tumor infiltration capacity.

## Author Contributions

EB and YS designed the work, wrote the manuscript, and prepared the figures. All authors drafted and revised the manuscript.

## Conflict of Interest

The authors declare that the research was conducted in the absence of any commercial or financial relationships that could be construed as a potential conflict of interest.
